# Supplementary material for: Composing games into complex institutions
Source: PLoS One. 2023 Mar 23;18(3):e0283361. doi: 10.1371/journal.pone.0283361 (PMC10035823; doi:10.1371/journal.pone.0283361)
Supplement: S1 File — (PDF) [file pone.0283361.s001.pdf]

Appendix I: Code vignette

In this Appendix we illustrate an example of using the *Haskell* implementation of compositional game theory in an interactive session from the development of Case #2.

Source code for replication and output displayed in the comments (`https://github.com/jules-hedges/open-games-hs/blob/729f5983bff7ee45ad8a2fac52300d728734e687/src/OpenGames/Examples/Governance/Irrigation.hs`)

No Monitoring

Analysis of no-monitoring game (source code)

```
irrigationNoMonitoring2Src = Block [] []
      [Line Nothing ["\"farmer1\"", "10", "Shirk"] ["dummy1"]
"irrigationStepMonitor" ["levelAfter1"] [],
      Line Nothing ["\"farmer2\"", "levelAfter1", "Shirk"]
["dummy2"] "irrigationStepMonitor" ["levelAfter2"] [],
      Line Nothing ["\"farmer3\"", "levelAfter2", "Shirk"]
["dummy3"] "irrigationStepMonitor" ["levelAfter3"] []]
      [] []
```

We provide the first case in more detail, the other cases should be understandable from the context.

```
> let a = Kleisli (\x -> certainly Crack)
> OpenGames.Engine.OpticClass.equilibrium irrigationNoMonitoring2
void ((a,()),(a,()),(a,()))
```

Agents in the farmer role can play **Crack** or **Flood**. If all players play **Crack** with certainty (responsibly “cracking” their gates open rather than irresponsibly flooding their fields regardless of the needs of downstream farmers) then we observe the following output:

```
[DiagnosticInfo {player = "farmer1", state = "()",
unobservableState = "(((),()),(\"farmer1\",10.0,False))", strategy
= "fromFreqs [(Crack,1.0)]", payoff = "2.0", optimalMove =
"Flood", optimalPayoff = "5.0"},DiagnosticInfo {player =
"farmer2", state = "()", unobservableState = "(((),8.0),
(\"farmer2\",8.0,False))", strategy = "fromFreqs [(Crack,1.0)]",
payoff = "2.0", optimalMove = "Flood", optimalPayoff =
"5.0"},DiagnosticInfo {player = "farmer3", state = "()",
unobservableState = "(((),(8.0,6.0)),(\"farmer3\",6.0,False))",
strategy = "fromFreqs [(Crack,1.0)]", payoff = "2.0", optimalMove
= "Flood", optimalPayoff = "5.0"}]
```

This is a non-empty list of strategies that dominate the input strategy, indicating that at least one player has incentive to deviate from their current strategy: that there is a strategy with higher payoff for each farmer.

In this alternative scenario, all players play **Flood** with certainty.

```
> let a = Kleisli (\x -> certainly Flood)
> OpenGames.Engine.OpticClass.equilibrium irrigationNoMonitoring2
void ((a,()),(a,()),(a,()))
```

We observe the following output:

```
[]
```

This represents an empty list. In the context of our engine this means that there is no deviating strategy. In other words, there is a Nash equilibrium in which all farmers "defect" by choosing **Flood** with certainty.

Lastly,

```
> OpenGames.Engine.OpticClass.equilibrium irrigationNoMonitoring2
void ((a,()),(a,()),((Kleisli (\x -> certainly Crack)),()))
```

Here the first two farmers play **Flood** with certainty; the last farmer `(Kleisli (\x -> certainly Crack))` plays **Crack** with certainty. Result:

```
[]
```

So this also constitutes an equilibrium. Under the *No Monitoring* regime there are equilibria whenever the top two farmers Flood regardless of what the tail-end farmer does. This is because only 2 of the original 10 units of water remain, commiting the tail-ender to receive 2 units regardless of what action they choose.

Monitoring Version 1 (three farmers + monitor)

This version adds an external monitor, who can **Work** and provide quality enforcement of a "no **Flood**" rule or **Shirk** by letting acts of **Flood** stand without penalty. Their choice of strategy is in turn influenced by whether they are paid a flat rate, a percentage of penalized farmer's proceeds, or some other scheme.

These and other schemes (monitor is not external but drawn internally from farmers) can all be implemented compositionally as minor variants on each other.

Monitoring game (source code)

```
irrigationMonitor3Src = Block [] []
      [Line Nothing ["\"monitor\"", "[Work, Shirk]", "()"] []
"dependentDecision" ["monitorWorks"] ["monitorWater1 +
monitorWater2 + monitorWater3 - if monitorWorks == Work then 1
else 0"],
```

```
      Line Nothing ["\"farmer1\"", "10", "monitorWorks"]
["monitorWater1"] "irrigationStepMonitor2" ["levelAfter1"] [],
      Line Nothing ["\"farmer2\"", "levelAfter1", "monitorWorks"]
["monitorWater2"] "irrigationStepMonitor2" ["levelAfter2"] [],
      Line Nothing ["\"farmer3\"", "levelAfter2", "monitorWorks"]
["monitorWater3"] "irrigationStepMonitor2" ["levelAfter3"] []]
      [] []
```

Convenience function for analysis

```
irrigationMonitor3Eq a b c d = equilibrium irrigationMonitor3 void
(Kleisli (const (certainly a)), (Kleisli (const (certainly b)),
()), (Kleisli (const (certainly c)), ()), (Kleisli (const
(certainly d)), ()))
```

Session with (`monitorPayRate = 0.2`, `punishmentRate = 0.7`)

```
> irrigationMonitor3Eq Work Crack Crack Crack
[]
> irrigationMonitor3Eq Shirk Flood Flood Flood
[DiagnosticInfo {player = "monitor", state = "()",
unobservableState = "((),())", strategy = "fromFreqs
[(Shirk,1.0)]", payoff = "0.0", optimalMove = "Work",
optimalPayoff = "2.0"}]
> irrigationMonitor3Eq Work Crack Crack Flood
[DiagnosticInfo {player = "farmer3", state = "()",
unobservableState = "(((),(Work,8.0,6.0)),
(\"farmer3\",6.0,Work))", strategy = "fromFreqs [(Flood,1.0)]",
payoff = "0.5000000000000004", optimalMove = "Crack",
optimalPayoff = "1.6"}]
```

Monitoring Version 2 (four farmers + monitor)

Monitoring game (source code)

```
> irrigationMonitor5Src = Block [] []
    [Line Nothing ["\"monitor\"", "[Work, Shirk]", "()"] []
"dependentDecision" ["monitorWorks"] ["monitorWater1 +
monitorWater2 + monitorWater3 + monitorWater4 - if monitorWorks ==
Work then 1 else 0"],
    Line Nothing ["\"farmer1\"", "8", "monitorWorks"]
["monitorWater1"] "irrigationStepMonitor2" ["levelAfter1"] [],
    Line Nothing ["\"farmer2\"", "levelAfter1", "monitorWorks"]
["monitorWater2"] "irrigationStepMonitor2" ["levelAfter2"] [],
    Line Nothing ["\"farmer3\"", "levelAfter2", "monitorWorks"]
["monitorWater3"] "irrigationStepMonitor2" ["levelAfter3"] [],
    Line Nothing ["\"farmer4\"", "levelAfter3", "monitorWorks"]
["monitorWater4"] "irrigationStepMonitor2" ["levelAfter4"] []]
    [] []
```

Convenience function for analysis

```
irrigationMonitor5Eq a b c d e = equilibrium irrigationMonitor5
void (Kleisli (const (certainly a)), (Kleisli (const (certainly
b)), ()), (Kleisli (const (certainly c)), ()), (Kleisli (const
(certainly d)), ()), (Kleisli (const (certainly e)), ()))
```

Output with (`monitorPayRate = 0.2`, `punishmentRate = 0.7`)

```
> irrigationMonitor5Eq Work Crack Crack Crack Crack
[]
> irrigationMonitor5Eq Work Crack Crack Crack Flood
[DiagnosticInfo {player = "farmer4", state = "()",
unobservableState = "(((),(Work,6.0,4.0,2.0)),
(\"farmer4\",2.0,Work))", strategy = "fromFreqs [(Flood,1.0)]",
```

```
payoff = "0.20000000000000018", optimalMove = "Crack",
optimalPayoff = "1.6"}]
> irrigationMonitor5Eq Work Crack Crack Crack Crack
[]
> irrigationMonitor5Eq Shirk Flood Flood Flood Flood
[DiagnosticInfo {player = "monitor", state = "()",
unobservableState = "((),())", strategy = "fromFreqs
[(Shirk,1.0)]", payoff = "0.0", optimalMove = "Work",
optimalPayoff = "2.7"}]
> irrigationMonitor5Eq Work Crack Crack Flood Flood
[DiagnosticInfo {player = "farmer3", state = "()",
unobservableState = "(((),(Work,6.0,4.0)),
(\"farmer3\",4.0,Work))", strategy = "fromFreqs [(Flood,1.0)]",
payoff = "0.40000000000000036", optimalMove = "Crack",
optimalPayoff = "1.6"},DiagnosticInfo {player = "farmer4", state =
"()", unobservableState = "(((),(Work,6.0,4.0,2.8)),
(\"farmer4\",2.8,Work))", strategy = "fromFreqs [(Flood,1.0)]",
payoff = "0.28000000000000025", optimalMove = "Crack",
optimalPayoff = "1.6"}]
```

Monitoring Version 3 (four farmers; last farmer is monitor)

Monitoring game (source code)

```
irrigationMonitor6Src = Block [] []
      [Line Nothing ["\"farmer4\"", "[Work, Shirk]", "()"] []
"dependentDecision" ["monitorWorks"] ["monitorWater1 +
monitorWater2 + monitorWater3 + monitorWater4 - if monitorWorks ==
Work then 1 else 0"],
      Line Nothing ["\"farmer1\"", "8", "monitorWorks"]
["monitorWater1"] "irrigationStepMonitor2" ["levelAfter1"] [],
      Line Nothing ["\"farmer2\"", "levelAfter1", "monitorWorks"]
```

```
["monitorWater2"] "irrigationStepMonitor2" ["levelAfter2"] [],
      Line Nothing ["\"farmer3\"", "levelAfter2", "monitorWorks"]
["monitorWater3"] "irrigationStepMonitor2" ["levelAfter3"] [],
      Line Nothing ["\"farmer4\"", "levelAfter3", "monitorWorks"]
["monitorWater4"] "irrigationStepMonitor2" ["levelAfter4"] []]
      [] []
```

Convenience function for analysis

```
irrigationMonitor6Eq a b c d e = equilibrium irrigationMonitor6
void (Kleisli (const (certainly a)), (Kleisli (const (certainly
b)), ()), (Kleisli (const (certainly c)), ()), (Kleisli (const
(certainly d)), ()), (Kleisli (const (certainly e)), ()))
```

Output (with `monitorPayRate = 0.2`, `punishmentRate = 0.7`)

```
> irrigationMonitor6Eq Work Crack Crack Crack Crack
    []
> irrigationMonitor6Eq Shirk Flood Flood Flood Flood
    [DiagnosticInfo {player = "farmer4", state = "()",
unobservableState = "((),())", strategy = "fromFreqs
[(Shirk,1.0)]", payoff = "0.0", optimalMove = "Work",
optimalPayoff = "3.0500000000000007"}]
> irrigationMonitor6Eq Work Crack Crack Crack Flood
    [DiagnosticInfo {player = "farmer4", state = "()",
unobservableState = "((),())", strategy = "fromFreqs
[(Work,1.0)]", payoff = "0.8000000000000003", optimalMove =
"Shirk", optimalPayoff = "2.0"},DiagnosticInfo {player =
"farmer4", state = "()", unobservableState = "(((),
(Work,6.0,4.0,2.0)),(\"farmer4\",2.0,Work))", strategy =
"fromFreqs [(Flood,1.0)]", payoff = "0.2000000000000018",
optimalMove = "Crack", optimalPayoff = "1.6"}]
```

Monitoring Version 4 (four farmers; last farmer is monitor; different water assignment)

Monitoring game (source code)

```
irrigationMonitor7Src = Block [] []
      [Line Nothing ["\"farmer4\"", "[Work, Shirk]", "()"] []
"dependentDecision" ["monitorWorks"] ["monitorWater1 +
monitorWater2 + monitorWater3 + monitorWater4 - if monitorWorks ==
Work then 1 else 0"],
      Line Nothing ["\"farmer1\"", "8", "monitorWorks"]
["monitorWater1"] "irrigationStepMonitor3" ["levelAfter1"] [],
      Line Nothing ["\"farmer2\"", "levelAfter1", "monitorWorks"]
["monitorWater2"] "irrigationStepMonitor3" ["levelAfter2"] [],
      Line Nothing ["\"farmer3\"", "levelAfter2", "monitorWorks"]
["monitorWater3"] "irrigationStepMonitor3" ["levelAfter3"] [],
      Line Nothing ["\"farmer4\"", "levelAfter3", "monitorWorks"]
["monitorWater4"] "irrigationStepMonitor3" ["levelAfter4"] []]
      [] []
```

Convenience function for analysis

```
irrigationMonitor7Eq a b c d e = equilibrium irrigationMonitor7
void (Kleisli (const (certainly a)), (Kleisli (const (certainly
b)), ()), (Kleisli (const (certainly c)), ()), (Kleisli (const
(certainly d)), ()), (Kleisli (const (certainly e)), ()))
```

Output (with `monitorPayRate = 0.2`, `punishmentRate = 0.7`)

```
> irrigationMonitor7Eq Work Crack Crack Crack Crack
    []
```

```
> irrigationMonitor7Eq Shirk Flood Flood Flood Flood
    [DiagnosticInfo {player = "farmer4", state = "()",
unobservableState = "((),())", strategy = "fromFreqs
[(Shirk,1.0)]", payoff = "0.0", optimalMove = "Work",
optimalPayoff = "6.199999999999999"}]
> irrigationMonitor7Eq Work Crack Crack Crack Flood
    [DiagnosticInfo {player = "farmer4", state = "()",
unobservableState = "(((),(Work,6.0,4.0,2.0)),
(\"farmer4\",2.0,Work))", strategy = "fromFreqs [(Flood,1.0)]",
payoff = "0.20000000000000018", optimalMove = "Crack",
optimalPayoff = "1.6"}]
> irrigationMonitor7Eq Work Flood Crack Crack Crack
    [DiagnosticInfo {player = "farmer1", state = "()",
unobservableState = "(((),Work),(\"farmer1\",8.0,Work))", strategy
= "fromFreqs [(Flood,1.0)]", payoff = "0.5000000000000004",
optimalMove = "Crack", optimalPayoff = "1.6"}]
```

Monitoring Version 5 (four farmers; first farmer is monitor)

Monitoring game (source code)

```
irrigationMonitor8Src = Block [] []
      [Line Nothing ["\"farmer1\"", "[Work, Shirk]", "()"] []
"dependentDecision" ["monitorWorks"] ["monitorWater1 +
monitorWater2 + monitorWater3 + monitorWater4 - if monitorWorks ==
Work then 1 else 0"],
      Line Nothing ["\"farmer1\"", "8", "monitorWorks"]
["monitorWater1"] "irrigationStepMonitor2" ["levelAfter1"] [],
      Line Nothing ["\"farmer2\"", "levelAfter1", "monitorWorks"]
["monitorWater2"] "irrigationStepMonitor2" ["levelAfter2"] [],
      Line Nothing ["\"farmer3\"", "levelAfter2", "monitorWorks"]
["monitorWater3"] "irrigationStepMonitor2" ["levelAfter3"] [],
```

```
      Line Nothing ["\"farmer4\"", "levelAfter3", "monitorWorks"]
["monitorWater4"] "irrigationStepMonitor2" ["levelAfter4"] []]
      [] []
```

Convenience function for analysis

```
irrigationMonitor8Eq a b c  d e = equilibrium irrigationMonitor8
void (Kleisli (const (certainly a)), (Kleisli (const
(certainlyb)), ()), (Kleisli (const (certainly c)), ()), (Kleisli
(const (certainly d)), ()), (Kleisli (const (certainly e)), ()))
```

Output (with `monitorPayRate = 0.2`, `punishmentRate = 0.7`)

(NOTE: In this case we are not only testing a given strategy, but searching for an equilibrium by brute force)

```
> filter (\(a,b,c,d,e) -> null (irrigationMonitor8Eq a b c d e))
[(a,b,c,d,e) | a <- [Work,Shirk], b <- [Crack,Flood], c <-
[Crack,Flood], d <- [Crack,Flood], e <- [Crack,Flood]]
[(Work,Crack,Crack,Crack,Crack)]
```

Monitoring Version 6 (four farmers; fourth farmer is monitor; wage and punishment)

Monitoring game (source code)

```
irrigationMonitor9Src = Block [] []
      [Line Nothing ["\"farmer4\"", "[Work, Shirk]", "()"] []
"dependentDecision" ["monitorWorks"] ["monitorPayoff wage pun c
monitorWorks"],
      Line Nothing ["\"farmer1\"", "9", "monitorWorks"] []
```

```
"irrigationStepMonitorNoTax" ["levelAfter1"] [],
      Line Nothing ["\"farmer2\"", "levelAfter1", "monitorWorks"]
[] "irrigationStepMonitorNoTax" ["levelAfter2"] [],
      Line Nothing ["\"farmer3\"", "levelAfter2", "monitorWorks"]
[] "irrigationStepMonitorNoTax" ["levelAfter3"] [],
      Line Nothing ["\"farmer4\"", "levelAfter3", "monitorWorks"]
[] "irrigationStepMonitorNoTax" ["levelAfter4"] []]
      [] []
```

Convenience function for analysis

```
irrigationMonitor9Eq a b c d e wage pun cost = equilibrium
(irrigationMonitor9 wage pun cost) void (Kleisli (const (certainly
a)), (Kleisli (const (certainly b)), ()), (Kleisli (const
(certainly c)), ()), (Kleisli (const (certainly d)), ()), (Kleisli
(const (certainly e)), ()))
```

Output with no punishment and transfer

```
> irrigationMonitor9Eq Work Crack Crack Crack Crack 0 0 0
[]
> irrigationMonitor9Eq Shirk Flood Flood Crack Crack 0 0 0
[DiagnosticInfo {player = "farmer4", state = "()",
unobservableState = "((),())", strategy = "fromFreqs
[(Shirk,1.0)]", payoff = "0.0", optimalMove = "Work",
optimalPayoff = "2.0"}]
> irrigationMonitor9Eq Work Crack Crack Crack Flood 0 0 0
[DiagnosticInfo {player = "farmer4", state = "()",
unobservableState = "((),())", strategy = "fromFreqs
[(Work,1.0)]", payoff = "1.0499999999999998", optimalMove =
"Shirk", optimalPayoff = "3.0"},DiagnosticInfo {player =
"farmer4", state = "()", unobservableState = "(((),
(Work,7.0,5.0,3.0)),(\"farmer4\",3.0,Work))", strategy =
```

```
"fromFreqs [(Flood,1.0)]", payoff = "1.0499999999999998",
optimalMove = "Crack", optimalPayoff = "2.0"}]
> irrigationMonitor9Eq Shirk Crack Crack Crack Flood 0 0 0
[DiagnosticInfo {player = "farmer1", state = "()",
unobservableState = "(((),Shirk),(\"farmer1\",9.0,Shirk))",
strategy = "fromFreqs [(Crack,1.0)]", payoff = "2.0", optimalMove
= "Flood", optimalPayoff = "5.0"},DiagnosticInfo {player =
"farmer2", state = "()", unobservableState = "(((),(Shirk,7.0)),
(\"farmer2\",7.0,Shirk))", strategy = "fromFreqs [(Crack,1.0)]",
payoff = "2.0", optimalMove = "Flood", optimalPayoff =
"5.0"},DiagnosticInfo {player = "farmer3", state = "()",
unobservableState = "(((),(Shirk,7.0,5.0)),
(\"farmer3\",5.0,Shirk))", strategy = "fromFreqs [(Crack,1.0)]",
payoff = "2.0", optimalMove = "Flood", optimalPayoff = "5.0"}]
```

Monitoring Version 7 (four farmers; first farmer is monitor; wage and punishment)

Monitoring game (source code)

```
irrigationMonitor10Src = Block [] []
      [Line Nothing ["\"farmer1\"", "[Work, Shirk]", "()"] []
"dependentDecision" ["monitorWorks"] ["monitorPayoff wage pun c
monitorWorks"],
      Line Nothing ["\"farmer1\"", "9", "monitorWorks"] []
"irrigationStepMonitorNoTax" ["levelAfter1"] [],
      Line Nothing ["\"farmer2\"", "levelAfter1", "monitorWorks"]
[] "irrigationStepMonitorNoTax" ["levelAfter2"] [],
      Line Nothing ["\"farmer3\"", "levelAfter2", "monitorWorks"]
[] "irrigationStepMonitorNoTax" ["levelAfter3"] [],
      Line Nothing ["\"farmer4\"", "levelAfter3", "monitorWorks"]
[] "irrigationStepMonitorNoTax" ["levelAfter4"] []]
      [] []
```

Convenience function for analysis

```
irrigationMonitor10Eq a b c d e wage pun costs = equilibrium
(irrigationMonitor10 wage pun costs) void (Kleisli (const
(certainly a)), (Kleisli (const (certainly b)), ()), (Kleisli
(const (certainly c)), ()), (Kleisli (const (certainly d)), ()),
(Kleisli (const (certainly e)), ()))
```

Output with no punishment and transfer

```
> irrigationMonitor10Eq Work Crack Crack Crack Crack 0 0 0
[]
> irrigationMonitor10Eq Shirk Flood Flood Crack Crack 0 0 0
[]
```

Output with punishment and transfer

```
> irrigationMonitor10Eq Work Crack Crack Crack Crack 0.1 0 0.2
[DiagnosticInfo {player = "farmer1", state = "()",
unobservableState = "((),())", strategy = "fromFreqs
[(Work,1.0)]", payoff = "1.9", optimalMove = "Shirk",
optimalPayoff = "2.0"}]
> irrigationMonitor9Eq Work Crack Crack Crack Crack 0.2 0 0.2
[]
```

Monitoring Version 8 (four farmers, rotating monitor)

Monitoring game (source code)

```
irrigationRotatingMonitorSrc = Block [] []
      [Line Nothing [] [] "nature (uniform [Left (Left (Left ())),
Left (Left (Right ())), Left (Right ()), Right ()])" ["switch"]
[],
      Line Nothing ["switch"] [] "irrigationFarmer1Monitor +++
irrigationFarmer2Monitor +++ irrigationFarmer3Monitor +++
irrigationFarmer4Monitor" ["discard"] []]
      [] []
```

Defining the rotating strategy

```
rotatingStrategy = (Kleisli (const (certainly Work)),
                    (Kleisli (const (certainly Crack)), ()),
                    (Kleisli (const (certainly Crack)), ()),
                    (Kleisli (const (certainly Crack)), ()),
                    (Kleisli (const (certainly Crack)), ()))
```

Output

```
> OpenGames.Engine.OpticClass.equilibrium
irrigationRotatingMonitor void ((), (((rotatingStrategy,
rotatingStrategy), rotatingStrategy), rotatingStrategy))
[]
```

Conclusion

The conclusion of this exercise is that, although several schemes can stabilize the cooperative **Crack** based equilibrium, or destabilize the uncooperative **Flood**-based equilibrium, no single approach to compensating or assigning the Monitor, or penalizing or disincentivizing **Flood**-ing can stably ensure cooperation in this sequential social dilemma, consistent with the embrace of

"institutional diversity", the thesis that real-world collective action institutions use a variety of systems and incentives to ensure successful cooperative resource management.
