# Supplementary material for: Composing games into complex institutions
Source: PLoS One. 2023 Mar 23;18(3):e0283361. doi: 10.1371/journal.pone.0283361 (PMC10035823; doi:10.1371/journal.pone.0283361)
Supplement: Attachment — Submitted filename: 2022_CompositionalGameTheory_FreyHedgesZahnTan_response.pdf [file pone.0283361.s003.pdf]

To Dr. Martinez-Garcia and *PLOSONE*,

Thank you for considering our original manuscript entitled "**Composing games into complex institutions**" (PONE-D-22-13128R1). Given the favorable reviews, and our understanding of the work's contribution, we would like to follow up on the editor's decision to reject.

> After careful consideration of the manuscript, we have concluded that it does not fully adheres to PLOS One publication criteria. Specifically, to be considered as a Methods manuscript, a submission must meet the criteria of Utility and Validation (https://journals.plos.org/plosone/s/submission-guidelines#loc-methods-software-databases-and-tools). To meet the validation criterium, your manuscript is missing a deeper preliminary analysis of the three cases considered.

If there are no concerns with the quality of the scholarship, and this decision is based entirely on PLOS ONE's formal guidelines of appropriateness, then we would like to appeal the decision on the grounds that this work does indeed satisfy the journal's Utility and Validation criteria.

The method we introduce in the manuscript does present a "prov[able] advantage over existing alternatives". We asserted this advantage in a way that would be clear to narrow domain specialists, but failed to communicate it suitably for PLOS ONE's more general audience. Recognizing and correcting this fault has already clearly improved the manuscript, and we hope that you will consider our revisions to improve how we communicate the work's contribution, and our validation of that contribution. (Our interpretation of the editor's concern is that we have not clearly communicated how our method improves over prior methods, and that more basic concerns—the usefulness of the method to the community, the long-term utility of the method—were not at issue.)

Where the original submission best describes our improvement over prior work is in these two sentences in the introduction:
"These new uses require a scalability, heterogeneity, and overall complexity that existing game design formalisms struggle to capture. Within familiar computational representations (typified by software libraries such as Gambit (24)), each additional player, choice, and stage added to a game contributes to an exponential growth of game outcomes, and a proliferation of equilibria."

However, these sentences require several things a general audience may lack, including
1. awareness of Gambit (and what it is, what it does, and how),
2. an appreciation for why explosive growth of outcomes and equilibria is bad.
We took our points to be self-evident, and the editor's concern shows us that this was a mistake. We also took our cases to be the "proof-of-principle experiments" required by the Validation criteria, but to a non-expert, our case implementations are not clearly proofs of principle, particularly with mere illustrations standing in for code.

The best existing alternative to our implementation of categorical game theory is Gambit, a package for computer-assisted design of economic games. Gambit is written around normal

("payoff matrix") and extensive form game representations. Our argument is that these representations are not adequate for complex games with large numbers of players, actions, and stages. However, we do not show it clearly enough in the manuscript.

We have remedied this with a new figure directly comparing our categorical with normal-form representations of all three games introduced in Case Study #2. The new figure shows that the three closely related game variants are directly comparable when represented categorically, and are harder to read, interpret, compare, or change when represented as payoff matrices.

As we elaborate in the figure caption, changes that are minor rewirings in the categorical description require new matrices in the normal form. We hope that the new figure, by presenting old and new representations side by side, makes it clear that our work "present[s] a proven advantage over existing alternatives" and "is an improvement over existing options" as shown by our "proof-of-principle experiment or analysis."

Going further, the process of developing the three cases involved adding several new technical features to our prior implementation of categorial game theory. Without these new features, Cases #2 and #3 would not have been possible to express. Those engineering advances were not the focus of the work (and we did not bring any attention to them in the submitted manuscript), but given the editor's concerns about Utility and Validity mentioning them helps us establish that this work represents a research advance, and not just a re-packaging or review of prior work.

Specifically, the technical features we developed while originally developing the three cases were:
* For case 2 we abstracted roles out of agents and developed two new operators for flexibly assigning roles to agents.
* For case 3 we developed a new operator for branching games. This feature involved several engine upgrades.
* We improved the syntax of our open games implementation for higher clarity, readability, and maintainability

We have added all of these clarifications and contributions to the manuscript. We hope by doing so that there is no further question that this work satisfies PLOS ONE's Utility and Validation criteria, and that the editor will find this work suitable for publication with PLOS.

Thank you for considering our appeal, and giving us the opportunity to show more clearly that our submission satisfies PLOS ONEs basic guidelines. For your convenience, here are the complete Utility and Validation criteria for submissions that present new methods, software, databases, and tools:

> Utility
> The tool must be of use to the community and must present a proven advantage over existing alternatives, where applicable. Recapitulation of existing methods, software, or databases is not

useful and will not be considered for publication. Combining data and/or functionalities from other sources may be acceptable, but simpler instances (i.e. presenting a subset of an already existing database) may not be considered. For software, databases, and online tools, the long-term utility should also be discussed, as relevant. This discussion may include maintenance, the potential for future growth, and the stability of the hosting, as applicable.
>
> Validation
> Submissions presenting methods, software, databases, or tools must demonstrate that the new tool achieves its intended purpose. If similar options already exist, the submitted manuscript must demonstrate that the new tool is an improvement over existing options in some way. This requirement may be met by including a proof-of-principle experiment or analysis; if this is not possible, a discussion of the possible applications and some preliminary analysis may be sufficient.
(From https://journals.plos.org/plosone/s/submission-guidelines#loc-methods-software-databases-and-tools)

Sincerely,
Seth Frey, Department of Communication, University of California Davis
Jules Hedges, Department of Computer and Information Sciences, University of Strathclyde
Joshua Tan, Department of Computer Science, University of Oxford
Philipp Zahn, Institute of Economics, University of St. Gallen
