# Supplementary material for: Composing games into complex institutions
Source: PLoS One. 2023 Mar 23;18(3):e0283361. doi: 10.1371/journal.pone.0283361 (PMC10035823; doi:10.1371/journal.pone.0283361)
Supplement: Attachment — Submitted filename: 2022_CompositionalGameTheory_FreyHedgesZahnTan_response.docx [file pone.0283361.s002.docx]

We thank the reviewers for their encouraging and helpful comments.

Reviewer #1: This paper provides an overview of compositional game theory and illustrates its applications using three case studies. The authors argue this theoretical framework, by allowing for modularity and abstraction, enables its users to build game-theoretic representations of more complex institutions than would be possible with traditional game theory.

The paper is generally well written. It is intended for an “audience beyond applied mathematics and theoretical computer science, with a particular focus on interdisciplinary and computational social scientists” (p.9). I believe this work would be of interest to not only these communities but also to environmental scientists and organizational economists, among others, as exemplified by the case studies.

My remarks are primarily about the scope and framing of the paper.

Major comments:

- There appears to be a slight discrepancy between the abstract/conclusion and the introduction as to what the main contribution of the paper is. The abstract and conclusion claim that the paper contributes the theoretical framework of compositional game theory (e.g., “Our contribution, compositional game theory, permits another approach of equally general appeal…”, p.7), whereas the introduction states that "[t]he primary aim of this work is to organize prior work on compositional game theory…” (p.9).

I believe the latter is a more accurate description of this paper. The authors have in fact made significant contributions to developing the theory of compositional game theory (e.g., refs 30-32, which include two authors of the current paper), but if I understand correctly, these contributions, including the mathematical formalism, are already published. It seems that the current paper instead reviews the key building blocks of this framework (Figures 1 and 2) followed by concrete applications to three case studies (Figures 3-5) and their implementations (Supplementary Materials)—which are certainly worthwhile contributions, particularly for practitioners and applied researchers interested in using the framework. I would suggest that the authors revise the framing along these lines.

Yes, this is right. We have updated the conclusion:

*Our contribution is to introduce computational social scientists to a theoretical framework for high-level game architecture, grounded in the mathematics of software engineering.*

- I find the introduction somewhat abstract. In particular, it is clear that there is interest in a compositional approach to game theory, but it is less clear when classical game theory fails.

In the abstract, we have more explicitly tackled the (sometimes sensitive) subject of where classical game theory fails and how the compositional approach improves.

*Relative to existing approaches in game theory, compositional game theory is especially promising for solving game systems with long-range dependencies, for comparing large numbers of structurally related games, and for nesting games into the larger logical or strategic flows typical of real-world policy or institutional systems.*

The software tutorial (Supplementary Materials, <https://github.com/philipp-zahn/open-games-hs/blob/master/Tutorial/TUTORIAL.md>) reads “NOTE: In its current form, compositional game theory is essentially a programmable reincarnation of classical game theory. The models you can generate with it can also be generated with classical game theory. What is different is how one approaches that modelling task: it becomes a programming task.” If this is in fact the main difference, I suggest that the authors highlight it explicitly in the main text. And if not, could the authors provide concrete examples of what compositional game theory can do but traditional game theory cannot? (I see attempts at this in the case studies, e.g., “Existing models often focus on one or two stages in isolation…” (p.12), but it’s unclear whether these are fundamental limitations of classical game theory or not.)

Construed broadly, classical game theory has no formal limitation that compositional game theory overcomes. Nevertheless, it has many practical limitations that compositionality renders elegant and simple. This is important because, as we argue, a good theorist often scopes the questions that are worth asking around the questions that are easy to ask. In this way, the compositional approach opens several subjects to more practical analysis: large chains of many games, comparisons of structurally similar games, complex logical/strategic flows through games (games of games), and the efficient interactive design of all of the above.

For example, in behavioral game theory studies of voting or organizational processes (e.g. Fishbacher, or Roberto Weber) it is increasingly common to see structures in which game outcome *x* doesn’t just provide payoff *w*, it triggers the play of game *y*, or activates player *z*. Under the classical approach, these logical extensions of game theory into systems of games require *ad hoc* notations, solvers, and forms of linkage. Under the open games approach of the compositional framework these extensions are practical, simple, and theoretically well-founded.

Similarly, the compositional approach does not solve the problem that large complexes of games may have dozens or hundreds of equilibria. Whether you are in classical or compositional game theory, counts of equilibria tend to grow linearly or super-linearly with the number of choices. But merely improving the visualization and designability of large games brings attention to the fact that they can be interesting and important even without tractable numbers of solutions.

Fast experimentation with alternatives is a boon to modeling, and a common practice in the design of simple games. Interacting on the timescales of thought with several varieties of a model is a very efficient way of discovering a design’s hidden tensions. Under compositional game theory, modelers and designers have a framework for exploring and iterating just as efficiently over arbitrarily large games.

We have added something of the above to the text. We have also strengthened the claim of the software tutorial materials to better describe open games in a way that is commensurate with their potential. The Tutorial text on GitHub now reads

*NOTE: The models we consider in this tutorial could be easily expressed in classical game theory. This is of course intentional as it (hopefully!) makes it easier to understand how to model using this engine. Moreover, a model represented in compositional game theory in theory often will be representable in classical game theory. The question is whether it is then practical to do so. In compositional game theory, it is natural to apply a divide-and-conquer strategy and cut the overall problem into parts, model the isolated parts and then address the connections between these parts. Like in programming more generally, this allows to deal with complex problems and enables the modelling of larger games. Lastly, the engine is not limited to game-theoretic analyses but can also be used in a broader sense, e.g. for simulation, modelling learning agents, and representing blends of systems comprised of strategic- and non-strategic components.*

Minor comments:

- Case 2 ends with a paragraph claiming that the authors “[could not] engineer a unique Nash equilibrium that is equitable, with all farmers extracting the same amount, without carefully combining several types of incentives” (p.14). Although I am inclined to believe this claim, I do not see any results/figures supporting it. I would suggest the authors provide at least a short verbal explanation for why this is the case or, preferably, some supplementary data showing how they arrived at this conclusion.

We have added the following text to the manuscript.

*The flexibility and extensibility of the compositional framework permitted us to come to this conclusion through a rapid and interactive exploratory design process. We started with the game of Figure 4b and ideas for possible extensions (including external monitoring, punishment, and peer monitoring with several kinds of incentives, all appearing in different combinations in the other panels). We then worked through the list of additional mechanisms, scanning single parameters for how they changed the base game’s equilibria (Appendix 1). Through this process we found that no single mechanism could succeed in stabilizing an outcome of equitable cooperation (and that defection by the head-ender is the most common pure strategy equilibrium).*

- "In compositional game theory, a player's actions include choices between games" (Figure 5, p.15). This is a neat feature of the compositional approach with applications to many other scenarios. Perhaps the authors could highlight it in the main text (in addition to the figure caption)?

Yes, we think so as well, and we now better emphasize the feature in the text. Nevertheless, its novelty is a bit hard to state with confidence. Game theory is so sprawling, and has been extended in so many ways, that it is actually possible to find dozens of works that have leveraged scenarios in which a player's actions include choices between games. Above in this response we mention the use of these mechanisms in behavioral game theory, and the text cites Hurwicz, Acemoglu et al., and Dziuda & Loeper. Nevertheless, our framework permits a general implementation of this feature that can standardize and accelerate its future use by others. Added text:

*Under the compositional framework, designers can nest games within each other, give players choices between games, and create complex logical flows across games or long-range dependencies within them.*

Reviewer #2: Overall Comments

In this paper, the authors present the framework of open games for formally representing complex game- theoretic interactions and provide three case-study examples illustrating the range of social-ecological systems and social institutions to which this framework can be applied. The paper starts with a short summary of the idea of open games and showing examples of how baseline games can be extended to describe complex institutions by composing games through methods including substitution, sequential and parallel composition, and branching, explaining how the formal string diagram representation of open games allows researchers to provide a compact representation of complex game architectures. By showing the examples of carbon certificate markets, irrigation monitoring schemes, and a principal agent model, the authors demonstrate how serial composition, branching, and careful examination of game architecture can be used to design and evaluate the role of institutions in the actions of game-theoretic agents.

Overall, I think that this is a useful paper that can help to advertise and communicate the open games framework as a useful tool for studying strategic dynamics in complex social systems. While compositional game theory has been studied in previous papers by the authors taking the approach of applied mathematics or theoretical computer science, this paper will serve as a useful introduction to practitioners in the social sciences who are interested in using systematic approaches to design and explore institutional structure in game theory. In addition, I think that this paper may be useful for applied mathematicians and others interested in the mathematical aspects of open games, as it can provide a tangible motivation for the conceptual framework before embarking on learning the mathematical details. The paper is also well-written overall and presents a useful illustration of the emerging field of compositional game theory, so I believe that this paper is appropriate for publication in PLoS One.

The reviewer’s summary gives us confidence that we are getting our meaning across. We have added the reviewer’s point that, in addition to helping social scientists, the paper could also motivate theorists to invest in developing the formalism.

*Among social scientists, calls for complex games have come from every discipline, but have been most clear and consistent from environmental science and organizational and institutional analysis. Within applied mathematics and mathematical game theory, this work motivates continued formal development by communicating the diversity and importance of its applications.*

However, I do think that the paper may benefit by including a bit more detail on the case study examples and the reasoning behind the form of string diagrams representing each model. I think that this will help further communicate the ideas of the paper for its intended audience. I also have a few minor comments regarding other aspects of the paper. Addressing some of the these points in a revised draft may help the final version of the paper more accessible to a wide audience of researchers interested in using this approach in applications.

Major Comment

In general, I believe that adding some additional exposition on the construction of the string diagrams for each example open game may be helpful for increasing the accessibility of this paper and the chance that readers may try to follow up on this paper by implementing compositional descriptions to model institutions relevant to their own research interest. Below, I have included a list of points that may be helpful to address in a revised version of the paper. Many of these points may only be an issue due to my own confusion, but these points may be helpful for increasing the readability for a broad audience.

• What do the circles and backwards triangles represent in the string diagram? From context, it seems like the triangles correspond to external inputs to games like institutions and players, and that the circles are used to represent when a single quantity serves as an input to two or more component games. While it may be feasible for readers to glean these aspects from context clues, I think it may be helpful to provide more description of general features of the string diagrams in the text to clarify how to read or construct representations for open games.

The reviewer gleaned correctly. To save our audience the same work we have improved the Figure 2 and Figure 3 captions (where these design elements are first introduced) to better highlight and describe them. One of our decisions for this manuscript has been to heavily load the captions, and put much of the burden on them for clarifying the images.

Still, as we elaborate below, a priority of this manuscript is to exhibit a computational framework, not a visualization paradigm. To manage the risk, from our past experience, that the visuals upstage their framework, we are directing readers to the manuscript dedicated to that visual language (see <https://arxiv.org/abs/1604.06061>) rather than giving them the depth of treatment that they received in that work.

• I think it may be helpful to explicitly mention places where the game compositions are used in the case study examples, such as the use of sequential composition in the three case studies and the use of branching in the Hurwicz principal agent model. This can help tie together the big picture idea of composing games presented in Figure 1 with the examples of designing games presented in the case studies.

To give readers a sense of how to expect these mechanisms of composability in action, we have added the following text to the Figure 1 caption:

*In the cases below, #1 and #2 use substitution, all three use sequential composition, #1 and #2 use parallel composition, and #3 uses branching.*

• In the irrigation model, is there a reason why the players are only represented externally to the module of game structure taking place in plot n (as represented in Figure 4, panel a)?

Yes. So in Figure 4 the module in panel A is the component that is being used in B, C. and D to represent the sequence of plots (it is used 3 times each in B and C and 4 times in D). By representing players external to this module it becomes possible to “plug” or “input” any player, depending on the context in which the module is called. If you think of function declarations in many languages, particularly compiled languages, each declaration defines the number and type of input, and the number and type of output. By analogy, the plot module that we show takes two required inputs, a player and a variable representing water level (and a third optional input used in panels C and D). It has a single output of the same water level type. Any player can be “entered” into the module, and if you look at how it is used throughout Figure 4, you will see that many different players are entered in, in very different ways. Under the hood, this metaphor to functions is not superficial. The work of developing compositional game theory was the work of grounding game theory in the mathematics that underlies programming. We added the following text to the caption of Figure 4:

*In a direct analogy to function declarations in many programming languages which define the types of the inputs and outputs, this module can be seen as a function, its inputs are a player, a water level parameter, and an optional penalty parameter, and its single output is a water level parameter. This module’s reuse in the other games, with different players entered in different ways, and water levels output from prior calls being used as the input to subsequent calls, all reflect the substance of the mapping from software design to institution design that compositional game theory permits.*

In particular, should the representation of the baseline actions always look like a version of the string diagram characterizing players and payoffs presented in Figure 2 (when modified for the appropriate numbers of players and actions)?

Figure 2 tries to illustrate a classic closed game in the visual lexicon of open games. So rather than representing an end state, it is actually supposed to represent the status quo beyond which this work is moving. To make this clear, we now use this figure to represent both a closed and open version of this game in different panels. Hopefully the issue is clear now. The new caption reads:

*Figure 2. Closed and open versions of the string diagram of an n-choice, two-player game. Two players emit decisions based on prospective information about (only) their own payoffs. Their decision feeds into the calculation of their own payoff and that of the other player. Time proceeds left to right, with players making decisions that emit payoffs. Arrows feeding backwards in time to players represent player preferences over future events. In these diagrams, specific choice sets and payoffs are abstracted away, improving parsimony as games scale. Formal string diagrams of this style map directly to game architectures in the sense that the computational representation of a game could be compiled to or from its diagram. Boxes are general, and can be used to represent players, payoffs, decision nodes, entire games, or any potential target of substitution. A. This “closed” version of a game is consistent with conventional game theory. Players are fixed, and results of the game feed back to those players. B. The closed game can be opened with the addition of inputs and outputs, represented by incoming or outgoing arrows. In the open version, players are replaced by inputs of player type, enabling flexibility as to how agents are selected to fill the player role, and reuse of the game in different contexts. This version of the game also has open outputs. In addition to feeding payoff information back to the players, it emits them as outputs that could, for example, be used to parameterize a downstream game. We show this in Figure 4, an irrigation social dilemma, which models the steady depletion of a water level variable by making the output of one decision unit the input of the next.*

Is the representation with players shown with a backward triangle in the irrigation model used because players can serve as both farmers and institutional monitors in different components of the open game? I feel that addressing some of these assumptions or justifications may be helpful for readers to understand why the example string diagrams take the form shown in the figures, and may help for interested readers to construct their own string diagrams for open games related to their own research.

Yes, this is right. We address the broader point below.

• In Figure 5, do the squares for landlord/work share or wage correspond to a zoomed out representation of a baseline game scenario with a players action and payoff (as considered in Figure 2)? If so, does such a representation correspond to the substitution operation presented in Figure 1? If the answer to either of these is yes, it is probably worth explaining that in the text. If the construction of this diagram depends on more subtle aspects of the modularity of representations of open games, I think it may be also be useful to mention this in order to highlight the rationale behind the construction of the string diagram.

In principle those squares could contain other games, but in this diagram they are primitive elements and there is not an intended connection to the structures in Figure 2. As it stands (and as we now say explicitly following the reviewer’s earlier advice), Figure 5 does not use substitution, just branching and serial composition. Hopefully our changes to Figure 2 and our other responses to these clarifying suggestions have been enough to make these diagrams clear to the reviewer. If during your next review you are able to articulate the misapprehension you had, this may help us more systematically spare other readers the same problem.

• How does the use of backward arrows representing preferences work in the different examples and composition rules? For example, what does a backward arrow mean for the different component games represented in Figure 1? In addition, when the farmers are represented by backwards triangles outside of the plot diagrams in Figure 4, should there be backward arrows running from the plot modules back to the triangles representing farmers? If not, why does the player represented by a triangle not have backward arrows corresponding to preferences over the actions in the plots, while players in Figure 2 have preferences over the payoffs? Clarifying this point may help readers in figuring out how they would design their own string diagrams.

The “plot” game depicted in panel A of Figure 4 generically represents the decision process of one abstract farmer. Note, that this component contains “farmer decision” which receives a backward arrow that connects the farmer’s decision to his/her payoff (like in Figure 2). The triangles “farmer 1”, “farmer 2” etc. are the identifying names of different players. These are non-strategic parameters used to instantiate copies of that generic decision process. The reason we parameterize this by the name becomes clear in panel D of the same figure: Here the same identity, e.g. farmer 1, is used in two different places of the game (the plot but also the “random draw”).

We also used the reviewer’s points to illustrate our point in Case 1 about long-range interactions between connected games:

*In this system, the allocation and resale stages are strategic (as signified by the presence of backwards-facing arrows, signifying a decision that depends on prospects about future interdependent outcomes). By contrast the production stages are non-strategic, in the sense that they can be made entirely on the basis of information that doesn’t depend on others. But once embedded between strategic decisions, production decisions begin to figure into a firm’s strategic reasoning.*

… and our point in Case 1 about the complexity of incentivizing fairness

*One thing that becomes apparent is that the process of structuring incentives toward greater fairness requires increasing the complexity of the institution and decisions within it. As indicated by the monitor’s backwards-facing arrows in Figs. 4C and 4D, the variations with greater capacity for fairness are also those with a greater number of interacting strategic decisions.*

We also further elaborated the Figure 2 caption to make the link between backwards-facing arrows and strategic reasoning explicit.

• Overall, I believe it would be helpful to include more text justifying the representation of different game architectures. I think this will help in making clear that the string diagrams are a formal method for representing open games, rather than more of an ad hoc approach for representing game interactions like informal flow charts. In addition, this may facilitate follow-up research in which readers can apply the approach of this paper to design their own game architectures and model different institutional incentives with game-theoretic modeling.

The reviewer’s questions and suggestions have been very useful for helping us into the minds of a fresh but interested audience. The clarifications these questions are inducing have already improved the accessibility of the work. Nevertheless, we do find that we are sticking more to the letter than the spirit of these suggestions. We have two major reasons for this. First, we have struggled in the past to make it clear that the primary advance of this work is not a visualization convention, but a mathematical framework (that makes the visualization convention possible, but only incidentally). Moreover, these representations are not "canonical" and cannot be used as the basis for general, mechanistic recipes to construct diagrams. Baked into each presented diagram is a particular modeler's viewpoint (ours), and nothing would stop us from giving a different viewpoint, for instance, zooming out in a different way. In that sense, one should think about one diagram more as a snapshot of the model. Since the diagrams are the most easily apprehended contribution, and so closely connected to the core contribution, we have observed that they often obscure or upstage it. So we have mostly restricted our clarity improvements to those that clarify the underlying framework, rather than the specifics of the visual lexicon.

Second, we do not intend that readers will construct string diagrams of their own, certainly not as end products. Ultimately, interested readers will construct their own computational specifications for open games, and will get string diagrams as a natural automatically generated representation of that specification. For those readers most inspired to develop this visual approach, the best we can do in this work is direct them to our team’s prior work fully developing the string diagram lexicon (see https://arxiv.org/abs/1604.06061).

Minors Comments

Here are a few additional minor comments which may be helpful to address in the revised manuscript.

• I am slightly confused by the statement in the first paragraph of the introduction that “this wildly successful research agenda obscured promising used of game theory for which equilibria are not the central concern”. There are large fields of research on non-equilibrium dynamics in game theory, including evolutionary game theory and models of learning in games. In addition, my understanding is that the open games framework is a clean way of representing more complex game interactions, but that the games that the framework aims to represent could be studied from an equilibrium framework or using other approaches like dynamical simulation of strategies. Given these factors, I think that the quoted statement in the introduction may be an unnecessary criticism of the existing state of game theory, and perhaps recentering the focus of the introduction on the representation of complex interactions may improve the introduction.

We have generalized the point to include non-equilibrium dynamics in the larger umbrella of “solutions”.

*However, this wildly successful research agenda has obscured promising uses of game theory for which equilibria and other solutions are not the central concern.*

It is true that games designed under the framework will still be formally studyable under a solution-focused perspective. Our argument is not that complex games are a break from conventional game theory because they don’t have solutions. They are a break from conventional game theory because they have too many solutions, a number that can scale exponentially depending on the solution type. At some point, complex games have too many solutions for equilibrium selection tools to make any sense of them. If there is still utility to compositional representations even when the idea of solving them isn’t useful, then our point stands.

It is important to stand by the point because a world in which large, complex, “descriptive” games are in the purview of game theory is one in which finding solutions is only one of several interesting things a person can do to a game. Recognizing the merit of this problem is part of recognizing the game theoretic merit of complex games. We clarify this point in the text:

*Construed broadly, classical game theory has no formal limitation that compositional game theory overcomes. The compositional approach does not solve the problem that large complexes of games may have dozens or hundreds of solutions. But merely improving the design of large games brings attention to the fact that they can be interesting and important without tractable numbers of solutions.*

• Is one benefit of the string diagram representation for open games that one can abstract away nonessential details from the specific game interactions, allowing to describe equivalence classes of game-theoretic interactions and institutions that impact strategic behavior? If so, this is probably useful to mention, as it may allow modelers to see connections between game architectures that have been stated in particular applied contexts but should actually have analogous equilibrium and dynamical behaviors.

Not string diagrams, but the formal system that makes diagrams possible, yes, precisely. This is one of the uses we have in mind when we discuss uses of game theory that are not centered around the idea of game solutions. We have borrowed a bit from the reviewer’s own description of this benefit to add the following to the introduction:

*Compositional game representations abstract nonessential details of specific games to allow systems of games to be compared, allowing modelers to see connections between game architectures, and to map differences in how they appear in social context to changes in their solutions.*
